# IL-8 promotes HNSCC progression on CXCR1/2-meidated NOD1/RIP2 signaling pathway

**DOI:** 10.18632/oncotarget.11445

**Published:** 2016-08-20

**Authors:** Leong-Perng Chan, Ling-Feng Wang, Feng-Yu Chiang, Ka-Wo Lee, Po-Lin Kuo, Chia-Hua Liang

**Affiliations:** ^1^ Graduate Institute of Clinical Medicine, Kaohsiung Medical University, Kaohsiung, Taiwan; ^2^ Department of Otolaryngology-Head and Neck Surgery, Kaohsiung Medical University Hospital, Kaohsiung Medical University, Kaohsiung, Taiwan; ^3^ Institute of Medical Science and Technology, National Sun Yat-Sen University, Kaohsiung, Taiwan; ^4^ Department of Cosmetic Science and Institute of Cosmetic Science, Chia Nan University of Pharmacy and Science, Tainan, Taiwan

**Keywords:** HNSCC, IL-8, NOD1, cancer progression

## Abstract

NOD1 (nucleotide-binding oligomerization domain 1) is overexpressed in head and neck squamous cell carcinoma (HNSCC) cells, as is IL-8 in cancer cells. However, the mechanism of the IL-8-mediated overexpression of NOD in HNSCC not been identified. This study determines whether IL-8 promotes tumor progression via the NOD signaling pathway in HNSCC. Higher IL-8, NOD1 and receptor-interacting protein kinase (RIP2) expressions were observed in HNSCC tissue than in non-cancerous matched tissue (NCMT), whereas NOD2 was weakly expressed. Furthermore, IL-8 stimulated the proliferation of HNSCC cells (SCC4, SCC9 and SCC25) but not dysplastic oral mucosa DOK cells. Exposure to IL-8 increased the clonogenicity of HNSCC cells. IL-8 siRNA inhibited cell proliferation and cell colony formation, suggesting that IL-8 is involved in HNSCC cancer progression. The expressions of CXCR1 and CXCR2 were higher in HNSCC tissue than in NCMT. HNSCC cells that were exposed to IL-8 exhibited higher expression of CXCR1/2 than did controls. The blocking of IL-8 by siRNA reduced CXCR1/2 expression in HNSCC cells, suggesting that the cancer progression of HNSCC cells that is induced by IL-8 depends on CXCR1/2. Additionally, IL-8 is associated with increased NOD1 and RIP2 expression and reduced NOD2 expression in three types of HNSCC cells. The blocking of IL-8 by siRNA reduces IL-8, NOD1 and RIP2 expressions in HNSCC cells, but not the level of NOD2. These results suggest that IL-8 has an important role in HNSCC progression via a CXCR1/2-meidated NOD1/RIP2 signaling pathway.

## INTRODUCTION

Head and neck squamous cell carcinoma (HNSCC) is the sixth most common cancer in the world. Oral cancer, predominantly oral SCC (OSCC), is a high-impact disease of the oral cavity. OSCC is generally detected in the late stages when the cancer has advanced, and therefore has a poor prognosis and survival rate [1]. Surgery and radiotherapy are currently the primary treatments, but they typically cause postoperative defects and functional impairments in patients owing to the location of OSCC in the head and neck [2]. Since morbidity and mortality rates associated with HNSCC have improved very little over the past 30 years, early detection or prevention of the disease is likely to be most effective. Therefore, basic research into HNSCC is increasing, focusing on the identification of specific biomarkers for the diagnosis of its nature and aggressiveness [3].

Cytokines are regulators of host responses to infection, inflammation, and trauma. Some cytokines make diseases worse (pro-inflammatory) while others reduce inflammation and promote healing (anti-inflammatory) [4]. HNSCC is known to develop many molecular strategies to escape from efficient anti-tumor immune responses. The tumor-induced T lymphocyte, granulocyte and neoangiogenesis responses in a local tumor microenvironment have been linked to increased growth and metastasis and reduced survival [5]. Researchers do not yet effectively understand the origin of the signals and mechanisms that underlie these responses. However, given the local and systemic nature of these responses, researchers hypothesize that SCC generates cytokines with pro-inflammatory, pro-angiogenic and immune-regulatory activities possibly contributing to the pathogenesis of HNSCC [6].

Interleukin-8 (IL-8) has a significant role in inflammatory responses in a tumor microenvironment; it affects tumor progression, metastasis and invasion, and so contributes to the pathogenesis of the disease [1]. IL-8 is responsible for most of the angiogenic activity that is induced by human OSCC [7]. Blocking IL-1 or TNF has been very successful in patients with rheumatoid arthritis, inflammatory bowel disease, or graft-vs-host disease but has not been successful in humans with sepsis [8]. Research has shown that IL-8 is a potential biomarker for OSCC in bodily fluids such as blood and saliva [9]. However, several studies have suggested that validation studies may have to be performed using a larger patient cohort and larger sample size to confirm conclusively IL-8 as a protein biomarker for OSCC [3, 5].

Recent studies have suggested that nucleotide binding oligomerization domain (NOD)-like receptors (NLRs) are key modulators in inflammatory diseases [10–11]. The current NLR signaling model posits that the caspase activation recruitment domain (CARD)-containing NOD proteins NOD1 and NOD2 interact with the CARD-containing receptor-interacting protein kinase (RIP2), activating the nuclear factor kappa B (NF-κB) pathway and mitogen-activated protein kinase (MAPK) pathways [12–14]. The NOD pathways directly regulate the release of cytokines, and particularly of pro-inflammatory cytokines, such as IL-1β, IL-6, IL-8 and other immune response-regulating cytokines such as IL-10 or TNF-α [15]. NLRs are expressed in various cell types, including macrophages, neutrophils, epithelial and endothelial cells, and dendritic cells, as well as in malignant tumors, including melanoma and HNSCC [16]. Genetic variations in NOD1 and NOD2 are associated with increased susceptibility to Crohn's disease [17]. The level of NOD1 receptors in gastric tumor tissues is regulated above that in paired non-tumor samples [18]. A previous study revealed that the expression profile of NLRs in HNSCC cells differed from that in healthy epithelial cells. Stimulation by NOD1 induced an immunological response in tumor cells that differed from that in normal epithelial cells [19]. The mechanism of the IL-8-induced NOD pathway from HNSCC and its regulation are poorly understood. Therefore, identifying its role in tumorigenesis can lead to novel treatment modalities for HNSCC.

## RESULTS

### Microarray, IL-8 and NOD signaling pathway

**Figure 1:**
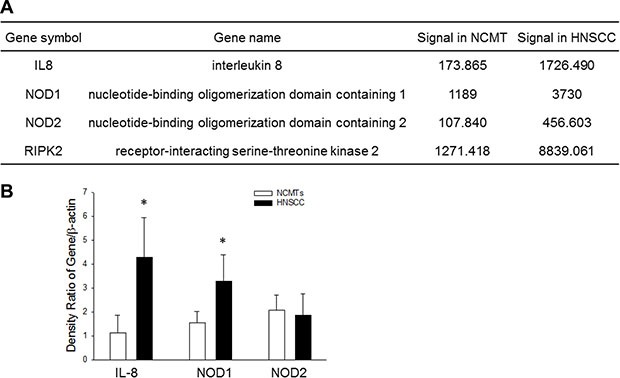
Expression of IL-8 and NOD signaling pathway in NCMT and HNSCC in human tissue (**A**) A microarray analysis to assess expression (fold change threshold > 2) of IL-8, NOD1, NOD2 and RIP2 in NCMT and HNSCC in human tissue. (**B**) Microarray data were verified by qRT-PCR: IL-8, NOD1 and NOD2 were upregulated in HNSCC relative to NCMT tissue (*n* = 6); *p < 0.05 indicates a significant difference from NCMT.

A previous study revealed significantly increased IL-8 levels in biopsied gingival tissue with periodontal inflammation. NOD signaling is increasingly associated with various conditions that are associated with chronic inflammation diseases [20]. Therefore, this study elucidated the effect of IL-8 on NOD signaling. A microarray analysis was firstly performed to elucidate the expression of innate immunity genes in HNSCC and NCMT in human tissue. Specifically, significantly increased expression (> 2-fold) was observed in several genes in the inflammasome signaling pathway in HNSCC, including IL-8, NOD1, NOD2 and RIP2, relative to NCMT. The increases were by a factor of 9.93 for IL-8, 3.14 for NOD1, 4.23 for NOD2 and 6.95 for RIP2 (Figure 1A). This relationship suggests that the upregulation of IL-8 correlates with the overexpression of NOD-mediated signaling in HNSCC.

### IL-8 and NOD1 activation in HNSCC

To study the potential role of IL-8 and NOD as informative biomarkers of HNSCC, revealed in the microarray study, the levels of IL-8, NOD1 and NOD2 in HNSCC and NCMT tissue (*n* = 6) were measured using qRT-PCR. As shown in Figure 1B, the mean IL-8 and NOD1 levels were statistically (3.70-fold and 2.12-fold, respectively) higher in patient tissue with HNSCC than in NCMT, whereas NOD2 was only weakly expressed. These results verify the use of IL-8 and NOD1 as biomarkers in HNSCC detection by microarray analysis (Figure 1A). Therefore, experimental data suggest that IL-8 and NOD1 had higher expression in HNSCC than in NCMT, whereas NOD2 was not expressed.

### IL-8 and NOD signaling pathway in HNSCC

**Figure 2:**
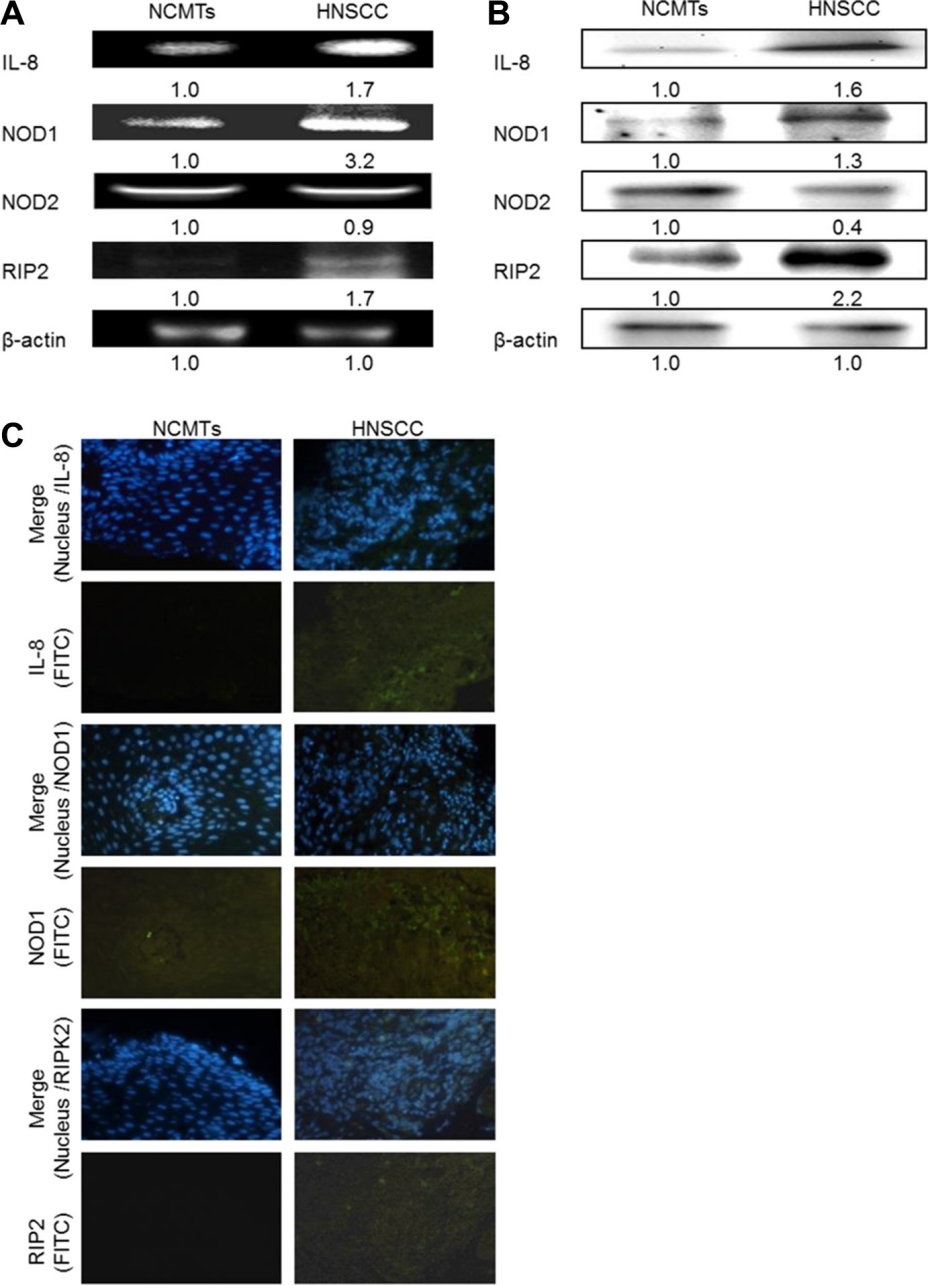
IL-8 and NOD signaling pathway in HNSCC IL-8, NOD1, NOD2, and RIP2 expressions in NCMT and HNSCC in human tissue were analyzed by RT-PCR (**A**) and western blotting (**B**). β-actin was used as internal control for sample loading. (**C**) Immunohistochemically-stained IL-8, NOD1 and RIP2 in NCMT and HNSCC viewed under an inverted fluorescent microscope (200× magnification).

NOD1 induces inflammation by stimulating cellular signaling pathways that involve the adaptor molecule RIP2. This process in turn induces NF-κB as well as p38 and JUN amino-terminal kinases (JNK) MAP kinases [21]. RT-PCR and western blotting confirmed that the IL-8 and NOD pathway-related gene and protein expressions (NOD1 and RIP2) were higher in HNSCC tissue than in NCMT, verifying the mRNA data. Similar results were obtained for IL-8, NOD1, NOD2 and RIP2 from RT-PCR and western blotting. Higher IL-8, NOD1 and RIP2 expressions were detected in HNSCC patient tissue than in NCMTs, whereas NOD2 was weakly expressed, according to RT-RCR (Figure 2A). Higher IL-8, NOD1 and RIP2 expressions were identified in HNSCC patient tissue than in NCMTs, but the levels of NOD2 were similar in both types of tissue, according to RT-RCR (Figure 2A). Up-expressions of IL-8, NOD1 and RIP2 and the down-expression of NOD2 protein in HNSCC patient tissue from those in NCMT were identified by western blotting (Figure 2B). Immunohistochemical staining was conducted to demonstrate the morphological localization of the IL-8, NOD1 and RIP2. Figure 2C shows greater immunostaining of IL-8, NOD1 and RIP2 in the HNSCC cytoplasm than in NCMT. Replacement of the primary specific antibody with the control (IgG) eliminated staining of all specimens (data not shown).

### IL-8 stimulates cell proliferation in HNSCC

**Figure 3:**
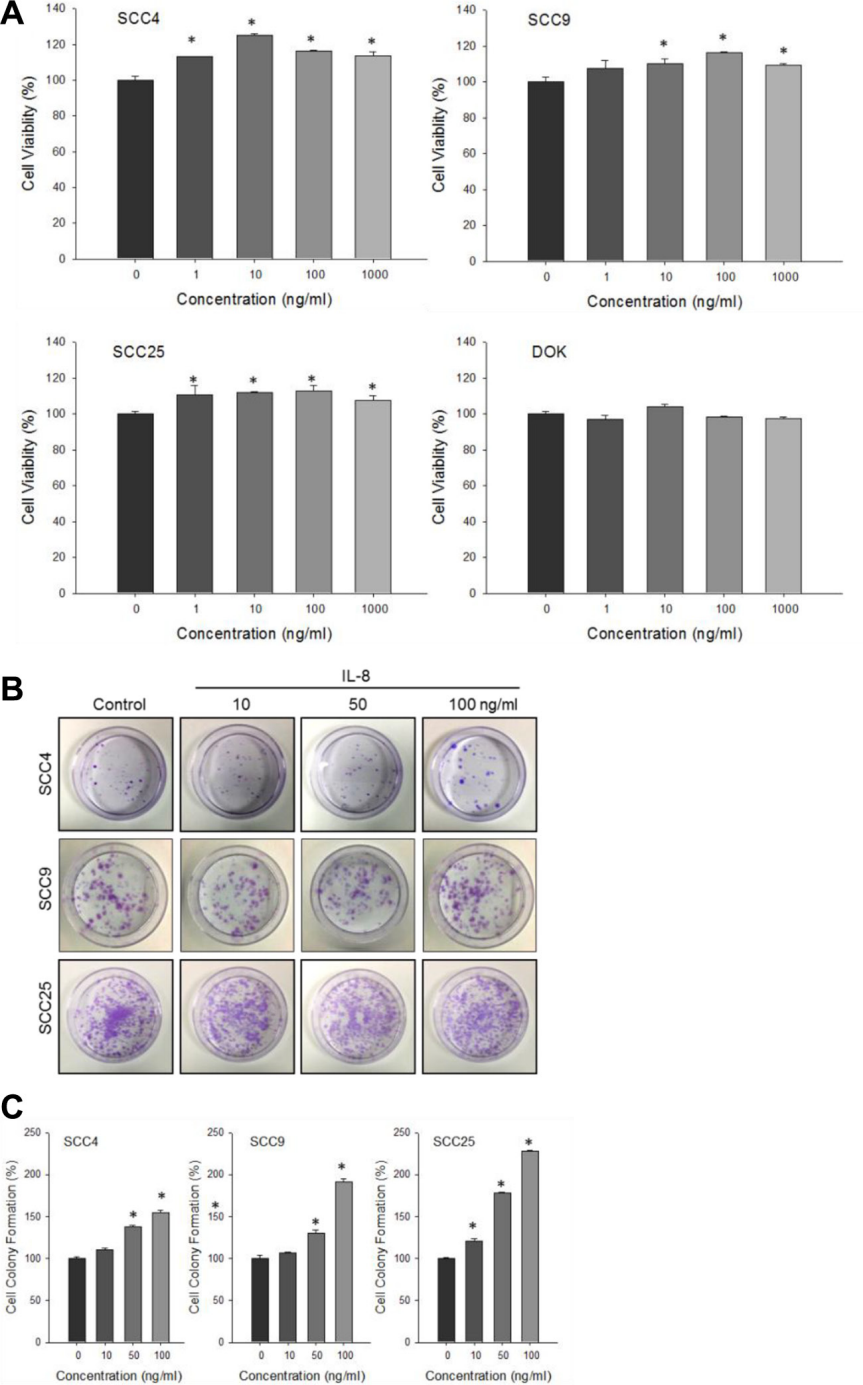
Effect of IL-8 on HNSCC cell proliferation (**A**) Human head and neck squamous cell carcinoma cells (SCC4, SCC9 and SCC25) and human dysplastic oral mucosa DOK cells were treated with IL-8 (0, 1, 10, 100 and 1000 ng/ml) for 72 h and cell proliferation was evaluated by MTT assay. Results are expressed as degree of cell proliferation relative to that of control. Number of colony-forming HNSCC cells following exposure to IL-8 (0, 10, 50 and 100 ng/ml) was calculated using clonogenic assay (**B**), and intensity of cell colony formation (**C**) was assessed. Each value is given as mean ± SD from triplicate experiments. *p < 0.05 implies a significant difference from control cells.

An earlier study cultured OSCC lines and tumor specimens suggested that the expression of IL-8 increases the pathogenicity of OSCC by providing a growth advantage [22]. To study the effect of IL-8 on HNSCC progression, the effect of IL-8 (0, 1, 10, 100 and 1000 ng/ml) on the proliferation of variously differentiated HNSCC cells (SCC4, SCC9 and SCC25 cells) and human dysplastic oral mucosa DOK cells was firstly determined. As shown in Figure 3A, IL-8 treatment of three types of HNSCC and DOK cell lines, at a concentration of 1–100 ng/ml, and especially at 10–100 ng/ml for 72 h slightly increased the proliferation of HNSCC cells, but not DOK cells. The results of *in vitro* clonogenic assays correlated strongly with those of *in vivo* assays of tumorigenicity in nude mice [23]. To study the effects of IL-8 on the relative clonogenicity, three types of HNSCC cell were treated with 10, 50 and 100 ng/ml of IL-8 for 3 h, and the cells were then allowed to form colonies for 14 days. The clonogenicity of HNSCC cells following exposure to IL-8 (Figure 3B) increased with their concentration. After treatment with 100 ng/ml of IL-8, SCC4, SCC9 and SCC25 cells exhibited 155.2%, 191.6% and 228.3% more intense cell colony formation than did the control cells (100%) (Figure 3C).

### Blockade of IL-8 reduced viability of NSCC cells

**Figure 4:**
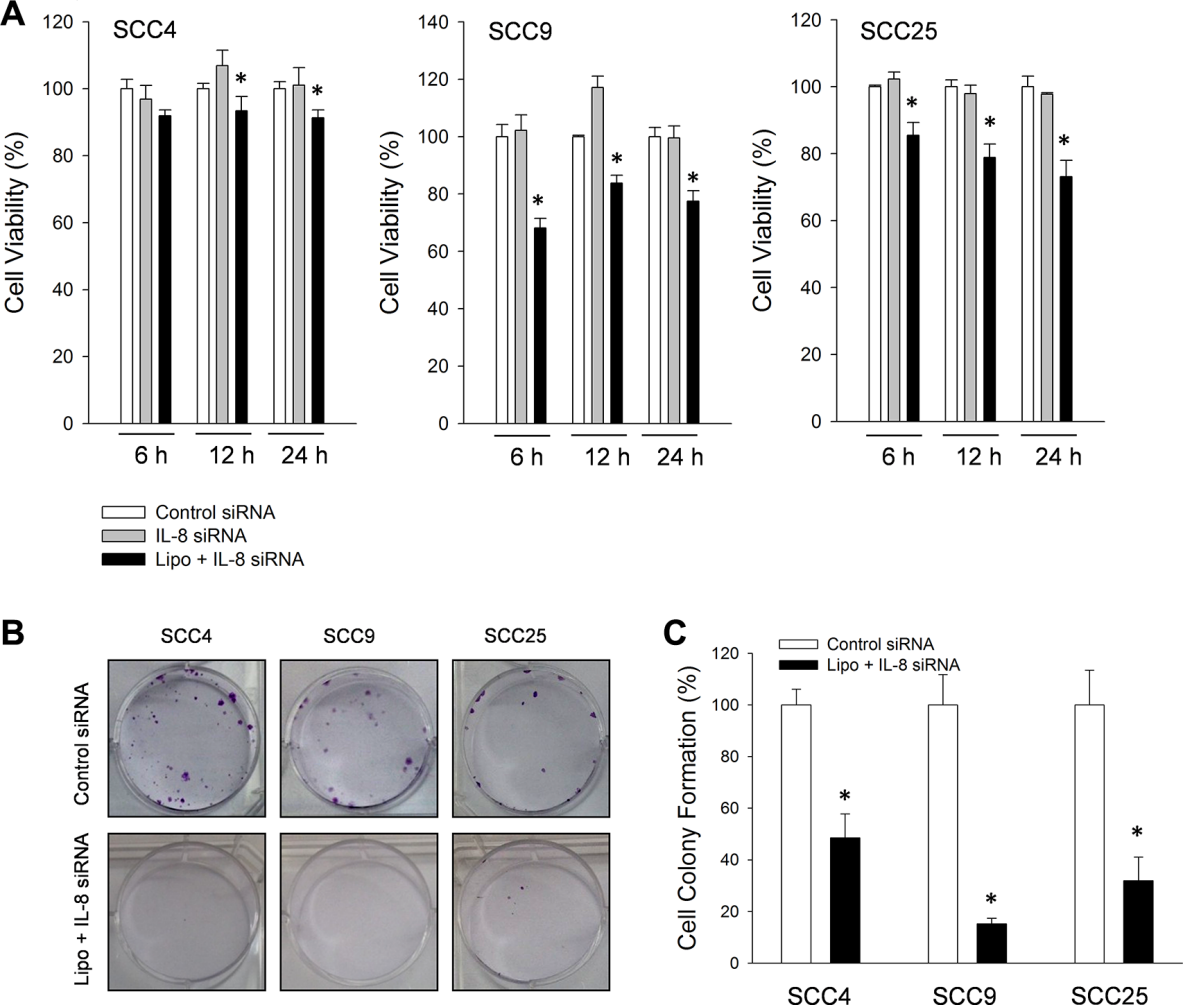
Knockdown of IL-8 reduced survival rate of three types of HNSCC cell (**A**) IL-8 (sc-39631) siRNA oligos (10 μM) were transfected into SCC4, SCC9 and SCC25 cells using Lipofectamine-2000, and these cells were then incubated for 6, 12 and 24 h before their viability was measured by MTT assay. Each value is mean ± SD from triplicate experiments. *p < 0.05 indicates a significant difference from cells treated with control siRNA. (**B**) Number of colony-forming HNSCC cells after exposure to control (sc-37007) siRNA and lipofectamine with IL-8 (sc-39631) siRNA (10 μM) for 6 h and then allowed to determine by clonogenic assay and the intensity of cell colony formation (**C**) was assessed.

The role of endogenous IL-8 was elucidated by using antisense oligonucleotides against human IL-8 mRNA (IL-8 siRNA). After HNSCC cells (SCC4, SCC9 and SCC25 cells) were incubated with IL-8 siRNA for 6, 12 and 24 h, the fall in IL-8 reduced cell survival of three types of HNSCC cell below that obtained using control IL-8 siRNA alone (Figure 4A). The following HNSCC cells were treated with IL-8 siRNA for 6 h and then allowed to form colonies for 14 days. IL-8 siRNA inhibited subsequent tumor cell proliferation and colony formation in soft agar in HNSCC cells, as shown in Figure 4B and 4C, indicating that IL-8 is involved in HNSCC progression.

### IL-8 upregulates expression of CXCR1/2 receptors in HNSCC

The biological effects of IL-8 are mediated through the binding of IL-8 to two cell-surface G protein-coupled receptors, CXCR1 and CXCR2 [24]. The potential for the induction of tumor cell proliferation by the enhanced expression of IL-8 by the HNSCC cells that express the related CXC receptors was evaluated.

**Figure 5:**
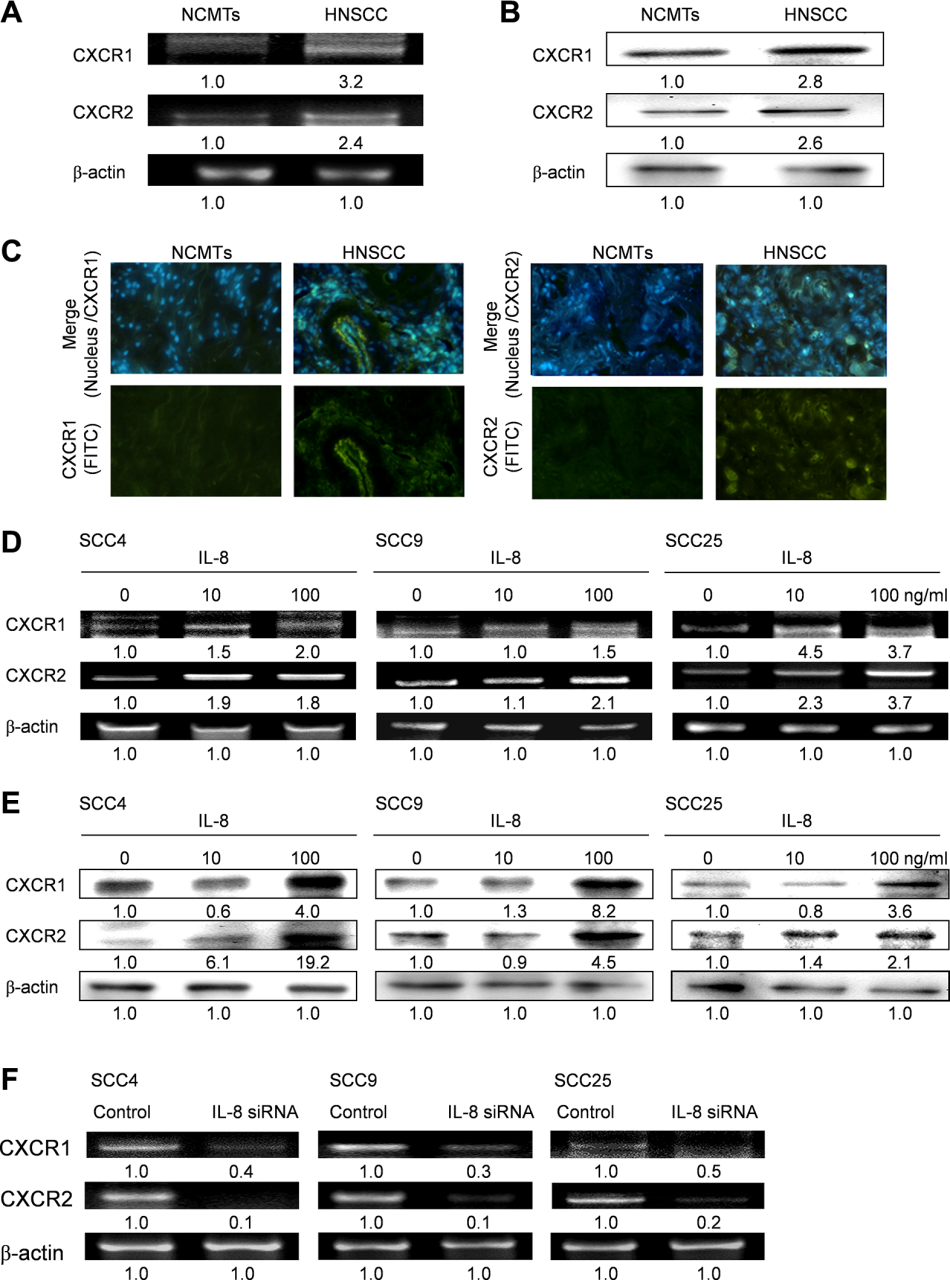
IL-8 signals through CXCR1 and CXCR2 in HNSCC (**A**) Levels of CXCR1 and CXCR2 in NCMT and HNSCC of human tissue were obtained by RT-PCR (A) and western blotting (**B**). (**C**) Immunohistochemically-stained CXCR1 and CXCR2 in NCMT and HNSCC viewed under an inverted fluorescent microscope (200× magnification). HNSCC cells (SCC4, SCC9 and SCC25 cells) were stimulated using IL-8 (0, 10 and 100 ng/ml) for 72 h and levels of CXCR1 and CXCR2 were obtained by RT-PCR (**D**) and western blot (**E**). (**F**) Knockdown of IL-8 by siRNA reduced expression of CXCR1 and CXCR2 in three types of HNSCC cell. Cells were treated with control siRNA and IL-8 siRNA for 6 h, and the amounts of CXCR1 and CXCR2 were obtained using RT-PCR.

The expression levels of CXCR1 and CXCR2 in HNSCC tissue and NCMT tissue were evaluated using RT-RCR (Figure 5A) and western blotting (Figure 5B), and expression levels in HNSCC were higher than in NCMT. As shown in Figure 5C, the expressions of CXCR1 and CXCR2 were greater in the HNSCC membrane than in NCMT, according to immunohistochemical analysis. Whereas IL-8 signals through corresponding chemokine receptors CXCR1 and CXCR2 in HNSCC cells, exposure of three types of HNSCC cells (SCC4, SCC9 and SCC25 cells) to IL-8 (10 and 100 ng/ml) for 72 h, the expressions of CXCR1 and CXCR2 were determined by RT-PCR and western blotting. As shown in Figure 5D and 5E, exposure of the three types of HNSCC cell to IL-8 increased the expressions of CXCR1 and CXCR2. Knockdown of IL-8 by siRNA reduced the expression of CXCR1 and CXCR2 in three types of HNSCC cell below that achieved using control siRNA (Figure 5F). Experimental results reveal that IL-8 upregulates CXCR1 and CXCR2 expression, suggesting that the cancer progression of HNSCC cells that is induced by IL-8 depends on both CXCR1/2 receptors.

### IL-8 activates NOD signaling pathway

**Figure 6:**
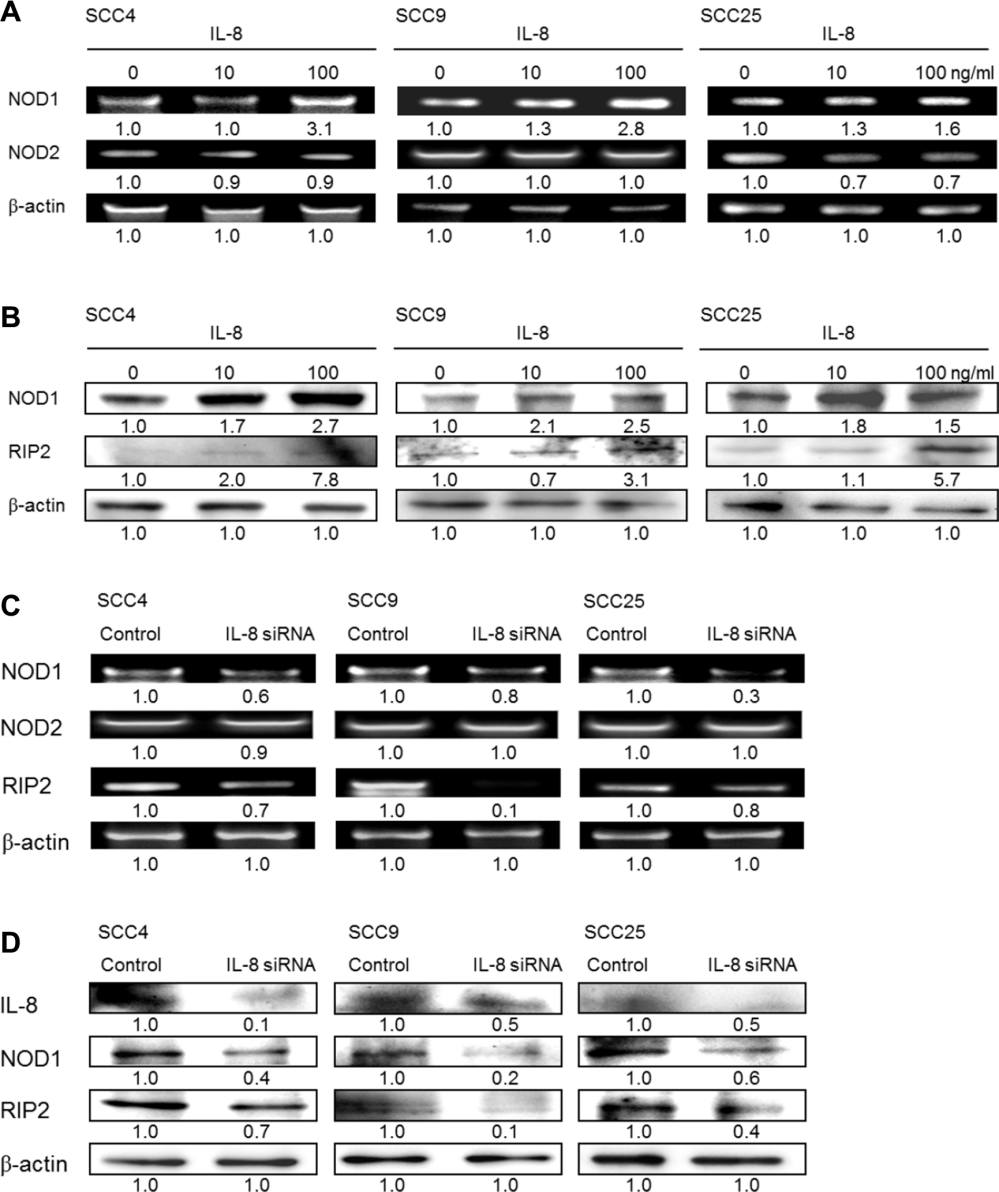
NOD1 signaling, but not NOD2, is involved in IL-8-mediated HNSCC cell proliferation (**A**) SCC4, SCC9 and SCC25 cells were treated with IL-8 (0, 10 and 100 ng/ml) for 72 h, and expressions of NOD1 and NOD2 were quantified using RT-PCR. (**B**) Three types of HNSCC cell were stimulated using IL-8 (0, 10 and 100 ng/ml) for 72 h, and then cell lysates were immunoblotted with an antibody specific for NOD1 and RIP2. (**C**) Cells were treated with 10 μM IL-8 siRNA for 6 h, and the amounts of NOD1, NOD2 and RIP2 were evaluated by RT-PCR. (**D**) HNSCC cells were exposure to control siRNA and IL-8 siRNA (10 μM) for 6 h, and then the expressions of IL-8, NOD1 and RIP2 were evaluated by western blot. b-actin was used as internal control for sample loading.

The effect of IL-8 in promoting tumor progression was measured from the NOD signaling pathway in HNSCC cells. To define the NOD-mediated signaling pathway that is associated with IL-8 treatment, the gene expressions of NOD1 and NOD2 in HNSCC cells of three types that were treated with IL-8 for 72 h were obtained by RT-PCR analysis. Figure 6A presents the experimental results, which reveal that the exposure of SCC4, SCC9 and SCC25 cells to IL-8 (10 and 100 ng/ml) increased NOD1 expression in a concentration-dependent manner. However, the expressions of NOD2 in the three types of HNSCC cell were unaffected or down-expressed by IL-8 treatment. Following treatment with IL-8 (10 and 100 ng/ml) for 72 h, protein expression of NOD1 and RIP2 activation was higher in SCC4, SCC9 and SCC25 cells than in control cells, as was confirmed by western blotting (Figure 6B). The role of IL-8 in regulating the NOD signaling pathway was verified using siRNA in HNSCC cells. The results revealed that the knockdown of IL-8 reduced NOD-mediated RIP2 activation in three types of HNSCC cell, while IL-8 siRNA treatment did not affect the expression of NOD2, according to RT-PCR (Figure 6C) and western blotting (Figure 6D). This finding suggests that the induction of HNSCC cancer progression by IL-8 is partially caused by the activation of CXCR1/2 and its NOD1 signaling partner RIP2.

## DISCUSSION

Previous reports have demonstrated that salivary IL-8 levels in oral lichen planus (OLP) patients are significantly reduced by treatment with dexamethasone, revealing that levels of IL-8 are affected by inflammation in OLP. Previous studies have revealed IL-8 at higher concentrations in the saliva of patients with OSCC than in controls, suggesting that IL-8 is a potential biomarker for oral cavity and oropharyngeal OSCC [7, 8]. IL-8 overexpression has been identified in tumor specimens of several human solid cancers, such as HNSCC [25]. Studies of tumor specimens and cultured HNSCC lines have verified that IL-8 expression may have a role in the pathogenicity of HNSCC by providing a growth advantage. Experimental data suggest that IL-8 was more strongly expressed in HNSCC than in NCMT.

Recent studies have shown that the promotion of tumor progression by IL-8 is primarily explained by its action as an autocrine growth factor for tumor cells, as well as an angiogenic factor [25, 26]. Adding an IL-8-neutralizing antibody slows the growth of human pancreatic cancer SG, FG and L3.3 cells by up to 10%, but this result is not statistically significant. Adding exogenous recombinant IL-8 increases the growth of three types of human pancreatic carcinoma cell by up to 20%. These data suggest that IL-8 is a minimal autocrine growth factor for these human pancreatic cancer cell lines [26]. Therefore, this study evaluates the effect of exogenous IL-8 on HNSCC progression. Experimental data reveal that the expression of IL-8 increased the proliferation of three differentiated HNSCC lines (SCC4, SCC9 and SCC25 cells) but not dysplastic oral mucosa DOK cells. IL-8 treatment promoted the formation of colonies of the hree cultured HNSCC lines. Blocking IL-8 by siRNA transfection effectively reversed HNSCC cell viability and cell colony formation, suggesting that IL-8 has a critical role in the progression of HNSCC.

CXCR1 and CXCR2 have a distinct ligand-binding pharmacology. CXCR1 receptors are activated only in response to the binding of IL-8 and granulocyte chemotactic protein-2. CXCR2 is activated by multiple CXC-chemokines, including growth-related oncogenes (GROα, β, and γ), neutrophil-activating peptide, and granulocyte chemotactic protein-2 [12]. Reports have shown that IL-8 has biological effects by binding to two chemokine receptors, CXCR1 (IL-8RA) and CXCR2 (IL-8RB), which are members of the seven transmembrane G-protein-coupled receptor (GPCR) family in human colorectal cancer [4]. Although CXCR1 and CXCRs have a considerable structural similarity, the proliferation and angiogenesis of human colon cancer cells probably depends on IL-8 binding only to CXCRs [27]. A previous study on the IL-8-mediated activation of the CXCR2 biological axis revealed that human neutrophil migration requires upregulation of the PI3k/Ras/Raf pathway [28]. The present work identified greater expression of CXCR1 and CXCR2 in HNSCC than in NCMT in human specimens. Treating three types of HNSCC cell directly with IL-8 significantly increased the expression of CXCR1 and CXCR2. The inhibition of IL-8 by siRNA reduced CXCR1 and CXCR2 expression, suggesting that activating IL-8 to both CXCR1/2 generates signals in HNSCC progression.

A previous study demonstrated that the endogenous IL-8 response to *Chlamydia* infections depends on NOD1 signaling via RIP2 as part of a signal system that requires multiple inputs for optimal IL-8 induction [21]. No clear links has been found between IL-8 and NOD activation in HNSCC. The tissue is needed to confirm the results, and to identify more the involvement of IL-8 and NOD in HNSCC. The present study was performed to test the hypothesis that the IL-8 level is elevated in the presence of chronic inflammation, irritation and the exposure of oropharyngeal mucosa to a carcinogen activating a NOD signaling pathway and thus contributing to a local environment that promotes the progression of these lesions to human HNSCC. Tests that involve human tissue microarray technologies have shown that various genes that are involved in the IL-8 and NOD signaling pathway are upregulated in HNSCC relative to NCMT. The expressions of IL-8, NOD1 and RIP2 were much higher in HNSCC than in NCMT, whereas the expression level of NOD2 was not. Therefore, we hypothesize the existence of an IL-8-mediated NOD1 signaling pathway on HNSCC progression. Treatment of three types of HNSCC cell by IL-8 increased NOD1 expression, but reduced or did not change the expression of NOD2. Knockdown of IL-8 by siRNA reduced NOD1 and NOD1-mediated RIP2 expression, but did not change NOD2 expression. A previous study demonstrated that the pharyngeal squamous cell carcinoma cell lines Detroit-562 cells showed prominent expression of NOD1, and FaDu cells exhibited presence of foremost NOD1, but expressed less NOD2 than did healthy primary human nasal epithelial cells [19]. Experimental data reveal that IL-8 has a key role in NOD1-mediated RIP2 activation and HNSCC progression.

The results herein reveal that IL-8 not only promotes the generation of HNSCC tissue, but also stimulates activity associated with the NOD pathway. Further validation studies may have to be performed using a larger sample cohort to provide a statistically comparison between HNSCC and healthy control groups and thereby to confirm the role of IL-8 and NOD1 as biomarkers of HNSCC. A comprehensive panel of markers that capture all tumors and low-cost, high-throughput technology would be ideal for early molecular detection for real-life screening. Therefore, adequately identifying the role of IL-8 in tumorigenesis could lead to novel treatment modalities for HNSCC.

## MATERIALS AND METHODS

### Patient selection and tissue samples

Patients were recruited from the Department of Otolaryngology-Head and Neck Surgery, Kaohsiung Medical University Hospital, Kaohsiung Medical University, Kaohsiung, Taiwan, over one year. Patients with documented T2N0M0 stage II squamous cell carcinoma of the oral cavity and oropharynx were recruited. All patients had recently been diagnosed with a primary disease, and had not received any treatment in the form of chemotherapy, radiotherapy or alternative remedies. All tissue samples were fresh-frozen in liquid nitrogen they underwent RNA and protein purification and microarray experiments; each was collected with the patient's consent with the approval of the institutional review boards of all participating institutions.

For immunohistochemical analysis, HNSCC and non-cancerous matched tissue (NCMT) were fixed in buffered formalin and embedded in paraffin, sliced into 3 μm thick sections, and mounted on glass slides. To isolate RNA and protein, HNSCC and NCMT tissue were placed in liquid nitrogen for further biochemical analyses. Flash-frozen tissue was removed from the freezer and placed into a mortar. The tissue was then ground to a fine powder and scraped into a liquid nitrogen-cooled microcentrifuge tube.

### Microarray

For the expression profiling of HNSCC and NSCT, total tissue RNA was extracted using Trizol reagent (Invitrogen, Carlsbad, CA, USA). An RNeasy Mini Kit (Qiagen, Hilden, Germany) was used to quantify the isolated. RNA isolated at OD 260 nm and a bioanalyzer (Agilent Technology, USA) was used to conduct a qualitative. A 0.2 μg mass of total RNA was amplified using a Low-Input Quick-Amp Labeling Kit (Agilent Technologies, USA) and labeled with Cy3 (CyDye, Agilent Technologies, USA) during the *in vitro* transcription process. A 0.6 μg mass of Cy3-labled cRNA was fragmented to a men size of approximately 50–100 nucleotides by incubation with fragmentation buffer at 60°C for 30 minutes. Correspondingly fragmented labeled cRNA was then pooled and hybridized using an Agilent Sure Print G3 Human V2 GE 8 × 60 K Microarray (Agilent Technologies, USA) at 65°C for 17 h. After they were washed and dried by blowing with a nitrogen gun, the microarrays were scanned using an Agilent microarray scanner (Agilent Technologies, USA) at 535 nm for Cy3. Scanned images were analyzed using Feature Extraction 10.5.1.1 software (Agilent Technologies, USA), which is image analysis and normalization software. The signal and background intensity were quantified for each feature.

### Quantitative real-time PCR (qRT-PCR) of IL-8 mRNA

qRT-PCR analysis was evaluate the mRNA expression of IL-8 and NOD1 in HNSCC and NCMT in human tissue to verify the results for the microarray (Welgene Biotech. Co., Taipei, Taiwan). Total RNA was extracted using a Total RNA Miniprep Purification Kit (GeneMark). qRT-PCR was conducted using specific primers 5′-GATTGAGAGTGGACCACACT-3′ and 5′-TCTCCCGTGCAATATCTACG-3′ for IL-8; 5′-GAGATTGGCTTCTCCCCTTC-3′ and 5′-CTGCCC AGGCTCTCGTTGCT-3′ for NOD1, and 5′-AGCCATTGT CAGGAGGCTC-3′, and 5′-CGTCTCTGCTCCATCA TAGG-3′ for NOD2. The amplification reactions were carried out in a 20 μl mixture, using SYBR Green Supermix (Bio-Rad Laboratories). After initial denaturation at 95°C for 3 minutes, 50 PCR cycles were performed at 60°C for 20s, t at 72°C for 20s, and at 83°C for 20s, followed by one minute at 95°C and a final minute extension at 55°C. All PCRs were normalized to internal control β-actin mRNA.

### RT-PCR analysis

To isolate tissue RNA, RNA was extracted from tissue using a Total RNA Miniprep Purification Kit (GeneMark). For cellular RNA extraction, RNAs were isolated from the vehicle control or cells that were treated with IL-8 at concentrations of 10 and 100 ng/ml for 72 h using the Trizol reagent (Invitrogen, Carlsbad, CA, USA). Single stranded cDNA was transcribed by priming with oligo-dT using SAMscript reverse transcriptase (GeneMark, Taipei, Taiwan). PCR amplification of the cDNA was performed in a reaction mixture that contained *Taq* polymerase (GeneMark, Taipei, Taiwan). The primers were as follows; IL-8 (150 bp), 5′-ACATACTCCAAACCTTTCCACCC-3′ and 5′-CAACCCTCTGCACCCAGTTTTC-3′; NOD1 (55 bp), 5′-TGATGCTGTTTCTGCCTCTC-3′ and 5′-AATTTG ACCCCTGCGTCTAG-3′; NOD2 (316 bp), 5′-GAATG TTGGGCACCTCAAGT-3′ and 5′-CAAGGAGCTTAGC CATGGAG-3′; RIP2 (559 bp), 5′-GCCCTCCTGTCCAG AGATTT-3′and 5′-TGGCAAATTCTTCTCCTTGAA-3′; CXCR1 (200 bp), 5′-GAGCCCCGAATCTGACATTA-3′ and 5′-GCAGACACTGCAACACACCT-3′; CXCR2 (202 bp), 5′-ATTCTGGGCATCCTTCACAG-3′ and 5′-TGCAC TTAGGCAGGAGGTCT-3′;and β-actin(295 bp), 5′-TCAC CCACACTGTGCCCATCTACGA-3′ and 5′-CAGCGGA ACCGCTCATTGCCAATCG-3′. The amplified RT-PCR products were electorphoresed on a 2% agarose gel, visualized by ethidium bromide staining and photographed under ultraviolet light. All results were normalized to the house-keeping β-actin gene. The obtained bands were visualized using Image J software.

### Western blot analysis

To extract protein from tissue, the frozen tissues were homogenized in lyses buffer (50 mM HEPES, pH 7.5, 150 mM NaCl, 10% glycerol, 1% Triton X-100, 1 mM EDTA, 1 mM EGTA, 50 mM NaF, 1 mM sodium orthovanadate, 30 mM *p*-nitrophenyl phosphate, 10 mM sodium pyrophosphate, 1 mM phenylmethylsulfonyl fluoride, 10 μg/ml aprotinin, and 10 μg/ml leupeptin). Supernatants were collected and analyzed. The cells (1 × 10^5^ cells/ml) were treated with 0, 10 and 100 ng/mL IL-8 for 72 h, and then washed using cold PBS and immediately lysed with lysis buffer. Tissue extracts and cell lysates were prepared and subjected to western blotting with antibodies against IL-8, NOD1, NOD2, RIP2, CXCR1 and CXCR2, respectively, as described elsewhere [29–31]. Proteins were visualized using ECL reagent and their relative expressions were determined by densitometry using the Image J software program, and normalized relative to β-actin.

### Immunohistochemical staining

Paraffin-embedded biopsies were performed using anti-IL-8, NOD1, RIP2, CXCR1 and CXCR2; immunohistochemical (IHC) followed. The primary antibody, diluted in phosphate buffered saline (1:400), was added to the tissue sections and incubated overnight in a moist chamber at 4°C; then corresponding secondary antibodies with FITC. Controls were established by incubating slides with an IgG isotype control rather than primary antibodies. The section was also stained with Hoechst33342 to study the nuclear morphology. The specific protein expressions and cell nuclei were examined using a fluorescence microscope (Nikon, TE2000-U, Japan).

### Cell culture and growth curve

Variously differentiated HNSCC cells (poorly differentiated SCC4 cells, moderately differentiated SCC9 cells and well differentiated SCC25 cells) were obtained from the American Type Culture Collection (Rockville, MD). Three cell lines were maintained in Dulbecco's modified Eagle medium (DMEM)/F12, which was supplemented with 0.4 μg/ml hydrocortisone (GIBCO, Grand Island, NY). Human dysplastic oral mucosa DOK cells were kindly supplied by Prof. Hann-Ming Sheu (National Cheng Kung University Medical College, Tainan, Taiwan). DOK cells were cultured in DMEM. All cells were cultured in a medium that was supplemented with 10% heat-inactivated fetal bovine serum (FBS) (Hazelton Product, Denver, PA, USA) and 1% penicillin-streptomycin at 37°C in 5% CO_2_. Onto each of numerous dishes with a diameter of 60-mm, 3 **×** 10^5^ cells were seeded and cultured for up to 72 h. At each assay point, cells were trypsinized harvested using a rubber policeman, washed with PBS, and counted under a microscope. Trypan blue staining was used to ensure that dead cells were not counted

### Cell proliferation and cell colony formation assay

Cells (1 × 10^5^ cells/ml) were treated for 72 h with 0, 1, 10, 100 and 1000 ng/ml IL-8 (MyBioSource, San Diego, California, USA) in F12 that contained 10% FBS. The control cells were treated with PBS. At the end of the assay period, cell viability was evaluated by colorimetric tetrazolium MTT [3-(4,5-dimethylthiazol-2-yl)-2,5-diphenyltetrazolium bromide] assay (Promega, Madison, WI) and absorbance was measured at 570 nm (BioTek, Synergy^TM^2). To determine long-term effects, cells were treated with 0, 10, 50 and 100 ng/ml IL-8 for 3 h. After the cells rinsed with fresh medium, they were allowed to form colonies for 14 days, which were then stained with crystal violet (0.4 g/l), as described elsewhere [23].

### Gene knockdown using small interfering RNA

The specific IL-8 (sc-39631) small interfering RNA (IL-8 siRNA, 10 μM) and control (sc-37007) siRNA (Santa Cruz, CA) were transfected into cells using the Lipofectamine RNAi MAX Reagent (Invitrogen), following the manufacturer's protocol. Cells were then incubated with control siRNA, IL-8 siRNA and IL-8 siRNA with lipofectamine for 6, 12 and 24 h before of cell viability and colony formation were measured and RT-PCR and western blotting assays were performed.

### Statistical analysis

The results are expressed as mean ± standard deviation (SD). Statistical differences were identified by one-way analysis of variance (ANOVA) followed by Dunnett's test or the Tukey-Kramer test. A *p value* of 0.05 was regarded as indicating significance. Data were analyzed and figures plotted using the relevant software (SigmaPlot Version 8.0 and SigmaStat Version 2.03, Chicago, IL).
